# Identification and characterization of the first cytokinin glycosyltransferase from rice

**DOI:** 10.1186/s12284-019-0279-9

**Published:** 2019-03-29

**Authors:** Pan Li, Kang Lei, Yanjie Li, Xingrui He, Shuo Wang, Renmin Liu, Lusha Ji, Bingkai Hou

**Affiliations:** 1 College of Pharmacy, Liaocheng University, Liaocheng, 252000 Shandong China; 2 College of Life Science, Shandong University, Qingdao, 250000 Shandong China

**Keywords:** Glycosyltransferase, Cytokinins, Gene cloning, Prokaryotic expression

## Abstract

**Background:**

Cytokinins are one of the five major hormones families in plants and are important for their normal growth and environmental adaptability. In plants, cytokinins are mostly present as glycosides in plants, and their glycosylation modifications are catalyzed by family 1 glycosyltransferases. Current research on cytokinin glycosylation has focused on the biochemical identification of enzymes and the analysis of metabolites in Arabidopsis. There are few studies that examine how cytokinin glycosylation affects its synthesis and accumulation in plants. It is particularly important to understand these processes in food crops such as rice (*Oryza sativa*); however, to date, cytokinin glycosyltransferase genes in rice have not been reported.

**Results:**

In this study, we identified eight rice genes that were functionally homologous to an Arabidopsis cytokinin glycosyltransferase gene. These genes were cloned and expressed in a prokaryotic system to obtain their purified proteins. Through enzymatic analysis and liquid chromatography-mass spectrometry, a single rice glycosyltransferase, *Os6*, was identified that glycosylated cytokinin in vitro. *Os6* was overexpressed in Arabidopsis, and the extraction of cytokinin glycosides showed that Os6 is functionally active *in planta*.

**Conclusions:**

The identification and characterization of the first cytokinin glycosyltransferase from rice is important for future studies on the cytokinin metabolic pathway in rice. An improved understanding of rice cytokinin glycosyltransferases may facilitate genetic improvements in rice quality.

**Electronic supplementary material:**

The online version of this article (10.1186/s12284-019-0279-9) contains supplementary material, which is available to authorized users.

## Background

Rice (*Oryza sativa*) is grown widely around the world and is the main food for more than 50% of the global population (Ramegowda et al. 2014). In recent years, with an increasing populations and environmental degradation, the land suitable for rice cultivation has diminished. To address these problems, the use of biotechnology to improve the stress resistance, yield and nutritional quality of rice is urgently required (Bansal et al. 2016). Cytokinins are important small molecule compounds, which are derivatives of adenine that occur naturally in plants (Plihalova et al. 2016). Cytokinins are one of the five major hormones found in plants, and are closely related to other plant hormones. Cytokinins affect plant growth and development (Zubo et al. 2017); including seed germination, chloroplast specialization, apical dominance, stress responses, cell differentiation and cell death. Therefore, the identification of the key enzymes of the cytokinin synthesis pathway in rice could be valuable for biotechnological applications. By functionally identifying these genes, their roles in the synthesis and metabolism of rice cytokinins, as well as in growth and environmental adaptation, could be further investigated. Better understanding these processes could lead to significant advances in the genetic improvement of rice yield and nutritional quality.

**Fig. 1:**
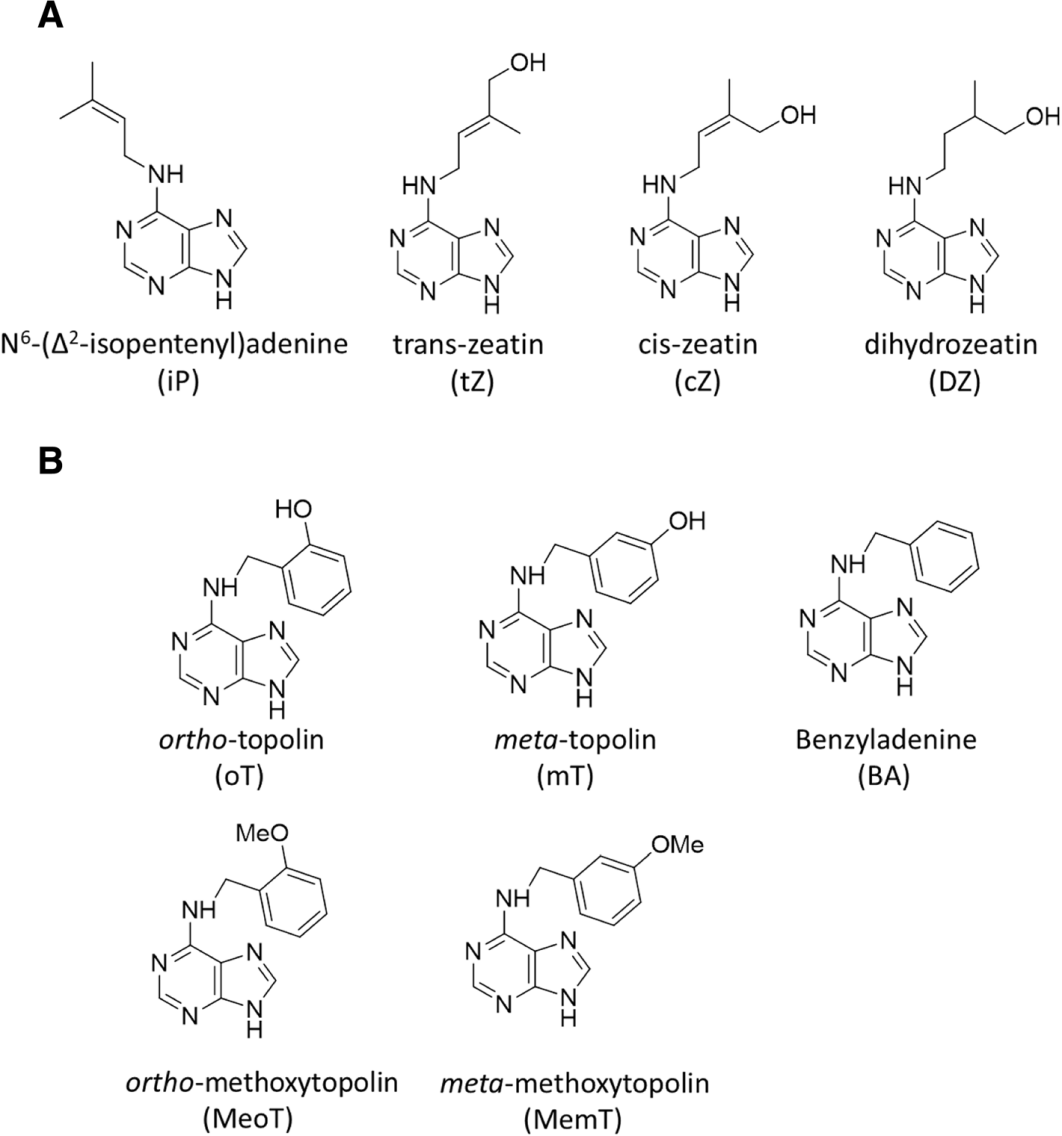
The molecular structure of common active cytokinins. **a** The molecular structure of Isoprenoid CKs. There are four forms of Isoprenoid CKs, N^6^-(∆^2^-isopentenyl) adenine (iP), trans-zeatin (tZ), cis-zeatin (cZ), dihydrozeatin (DZ). **b** The molecular structure of Aromatic CKs. There are five forms of Aromatic CKs, *ortho*-topolin(oT),*meta*-topolin(mT),benzyladenine(BA),*ortho*-methoxytopolin(MeoT), *meta*-methoxytopolin(MemT)

The first naturally occurring cytokinin identified in plants was zeatin, which was obtained from unripe corn endosperm (Frebortova et al. 2017). Later, as cytokinin research progressed, more and more types of cytokinins were identified. The naturally occurring cytokinins in plants can be divided into isoprenoid and aromatic forms on the basis of the type of cytokinin side chain. The molecular structures of the common active cytokinins were shown in Fig. 1 (Xu et al. 2017).

There are many cytokinin synthesis pathways in plants (Yamburenko et al. 2017). The pathways that have been identified to date can be divided into the transport RNA (Morrison et al. 2015) and de novo synthesis (Ciaglia et al. 2017). The transport RNA pathway produces too few cytokinins to meet the growth needs of plants. Therefore, the de novo synthesis pathway is the generally accepted main pathway for cytokinin synthesis. The de novo synthesis pathway also includes the adenosine monophosphate (AMP) pathway, the ATP/ADP pathway, and the alternative pathway. In the AMP pathway, dimethyl propylene diphosphate (DMAPP):AMP isoprenyltransferase catalyzes the transfer of an isopentyl group from dimethyl propylene diphosphate to the N6 position of AMP to produce a cytokinin with active pentyl adenosine-5-phosphate (iPMP) and isoamyladenosine groups (Ashihara et al. 2018). In the ATP/ADP pathway, the IPT4 enzyme obtained from *Arabidopsis thaliana* can preferentially utilize ATP and ADP as substrates in the presence of ATP, ADP and AMP. Isoprenoid adenosine-5-triphosphate and isoamyladenosine-5-diphosphate are possible reaction products of cytokinins, and can form zeatin through a further one-step hydroxylation reaction (Yu et al. 2015). The alternative pathway is an iPMP-independent pathway that transfers the hydroxylated side chain to the N6 site of adenine. This pathway was identified after it was discovered that the precursor of trans-zeatin nucleoside phosphate was not primarily derived from iPMP (Kaltenegger et al. 2018).

To date, studies of the cytokinin metabolic pathway have focused on Arabidopsis. The cytokinin receptors are three histidine kinases, AHK2, AHK3 and CYTOKININ RESPONSE 1 (also known as AHK4) (Lomin et al. 2015). There are also more than 20 response regulators that are associated with cytokinins. These Arabidopsis response regulators can be broadly classified into four types: A-type, B-type, and C-type as well as Arabidopsis pseudoresponse regulators (Kurepa et al. 2014). In addition, the degradation of cytokinin mainly depends on the activity of cytokinin oxidative dehydrogenase, which irreversibly inhibits cytokinin degradation (Yeh et al. 2015; Xiao et al. 2016). Morris et al. (1999) found that cytokinins are extensively degraded in the anthers and pollen of cytokinin oxidative dehydrogenase transgenic corn, which eventually leads to male sterility during spore formation.

In nature, cytokinin compounds are found mostly as glycosides. Various cytokinin glycosides can be formed owing to differences in sugar types, attachment positions and aglycones. Further modification of these groups produces a wide variety of cytokinin compounds in nature and glycosylation is particularly important for this diversity (HIuska et al. 2016). Cytokinin glycosylation is mainly catalyzed by family 1 glycosyltransferases (GT1s) (Lao et al. 2014), which are conserved across different plant species. GT1s transfers the active sugar donor, usually a UDP-glycosyl group, to the hydroxyl group of the substrate to form a cytokinin glycoside product.

The cytokinin glycosides are still poorly understood. However, cytokinin glycosyltransferase genes have been studied in Arabidopsis. For example, Hou et al. (2004) used molecular and biochemical methods to demonstrate that the Arabidopsis glycosyltransferase UGT76C2 catalyzes cytokinin glycosylation in vitro. There are many substrates that can be catalyzed by glycosyltransferases, including free-form trans-zeatin, cis-zeatin, dihydrozeatin, isopentenyl adenine, 6-benzyl adenine and kinetin. After glycosylation, these mitogens form their corresponding N-glycosides. In 2011, Wang et al. (2011) conducted experiments in vivo using *Arabidopsis thaliana* as the model. They showed that UGT76C2 catalyzes cytokinin glycosylation in plants and participates in the regulation of cytokinins. The modifications made to cytokinins by glycosyltransferases may be considered as “fine tuning” the synthesis, metabolism and function of these compounds. Glycosylation may affect the transport of cytokinins in plants, the normal growth and development of plants, the specific distribution of cytokinins in tissues and cells, signal transduction processes associated with these molecules, and upstream regulatory factors. Therefore, studying cytokinin glycosylation is of great significance for understanding the metabolic regulation of cytokinin compounds and for clarifying their physiological effects.

Studies of cytokinin glycosyltransferase genes in rice have not previously been reported. Here, we selected eight candidate rice glycosyltransferase genes and found that one of these, *Os6*, encodes a protein that can glycosylate cytokinin in an in vitro enzymatic reaction, as determined by liquid chromatography-mass spectrometry (LC-MS). We transformed Arabidopsis with Os6 to obtain homozygous transgenic lines overexpressing *Os6*. We examined these plants for growth and developmental phenotypes, as well as analyzing their cytokinin synthesis, metabolic and signal transduction pathways. The candidate gene selected in this project, *Os6*, is the first rice gene reported to glycosylate cytokinin. This work lays a good theoretical and practical foundation that, with the ongoing study of Os6, will improve our understanding of cytokinin glycosyltransferase functions in rice. This should be beneficial for future genetic breeding approaches to improve rice quality.

## Results

### Screening and identification of rice cytokinin glycosyltransferase candidate genes

**Fig. 2:**
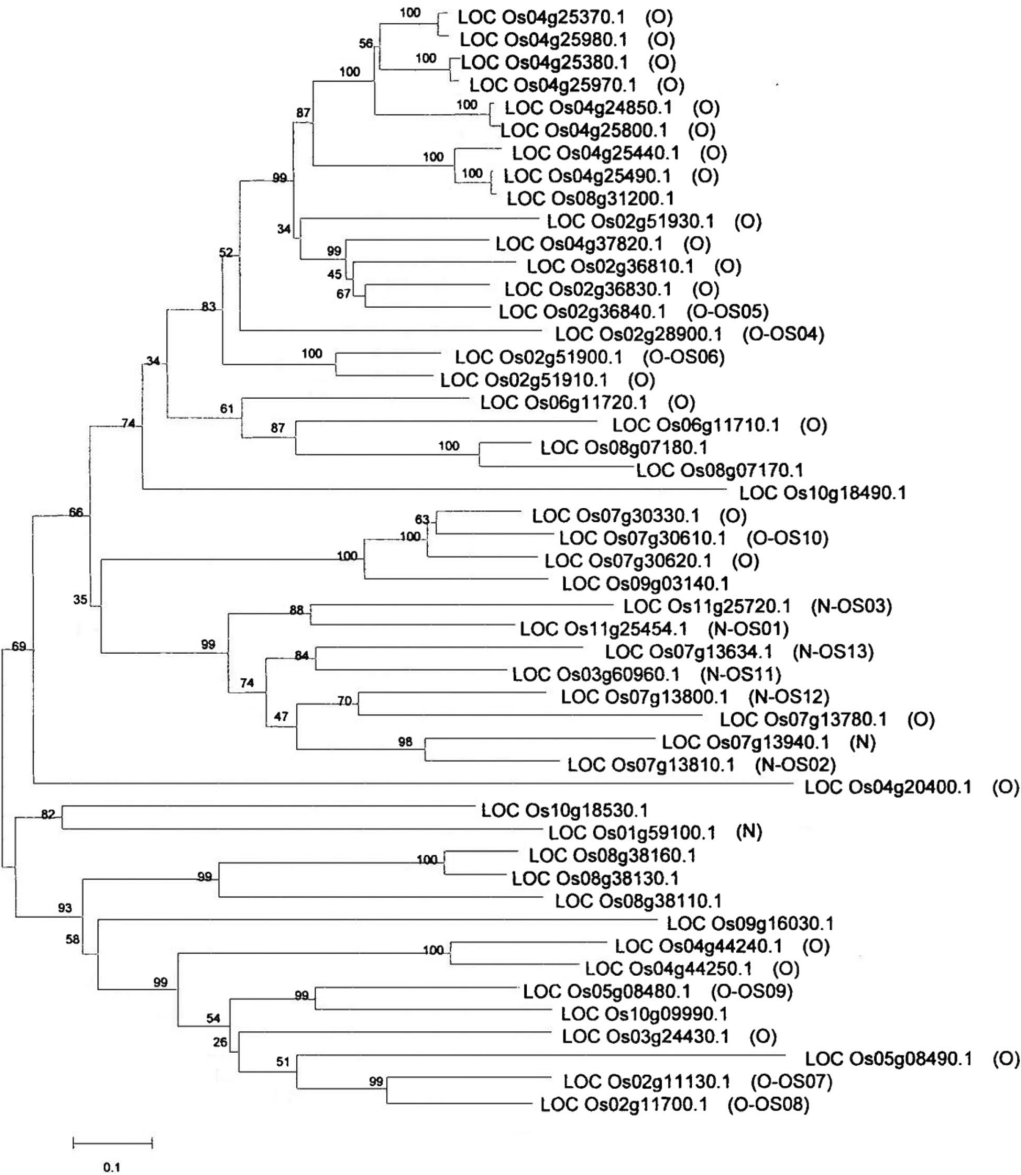
Rice cytokinin glycosyltransferase phylogenetic tree. The phylogenetic tree was constructed using MEGA software based on the similarity information of these sequences

The complete rice genome of rice in now available through the rice genome database (http://rice.plantbiology.msu.edu). In 2008, Cao et al. constructed a phylogenetic tree of glycosyltransferases in rice using bioinformatics methods and identified 609 potential glycosyltransferase genes. Among them was GT1, which encodes the enzyme that transfers UDP-glucose. According to Cao’s prediction, the family GT1 contains the largest number of genes (Cao et al. 2008). There are 8 and 41 genes with glycosyltransferase activity at the N- and O- positions, respectively, which may represent glycosylated cell classifiers, and the phylogenetic tree was constructed using MEGA software based on the similarity information of these sequences (Fig. 2). In our study, we selected eight candidate genes capable of glycosylating cytokinins, LOC_Os01g59100 (*Os6*), LOC_Os02g11130, LOC_Os07g30330, LOC_Os02g11700, LOC_Os07g30610, LOC_Os07g13800, LOC_Os11g25454 and LOC_Os07g13810. LOC_Os01g59100 corresponded to the gene referred to as *Os6* in this study.

### Spatiotemporal expression pattern of *Os6* in WT rice and *Os6* cloning

**Fig. 3:**
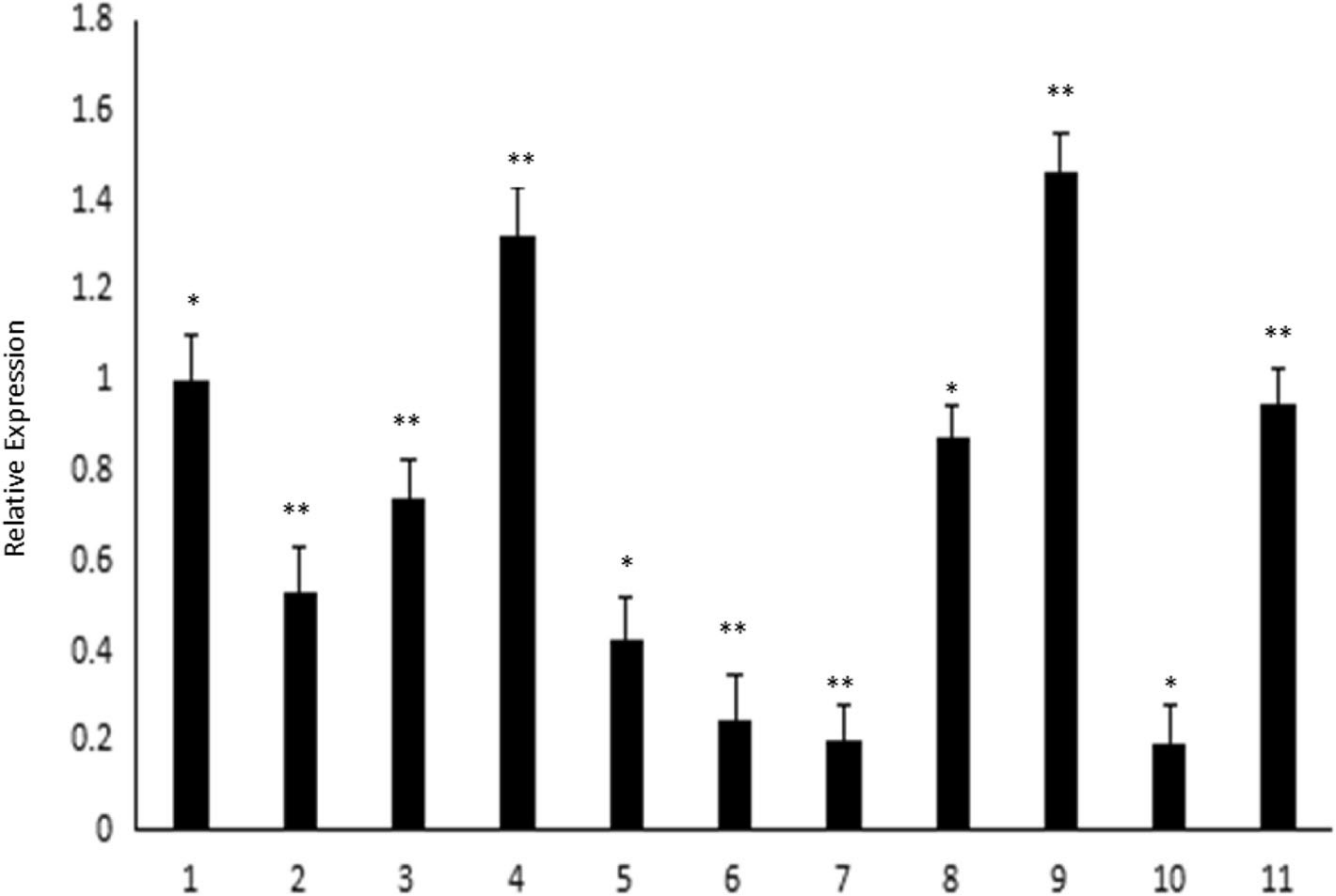
Time and space expression pattern of *Os6.* In order to further understand the expression pattern of *Os6*, RNA was extracted from different tissues and organs of the wild-type rice plants, then Reverse transcription into cDNA, detection of *Os6* expression pattern by Real-Time PCR. 1: Seedling Root; 2: Seedling Stem; 3: Seedling Leaves; 4: Seedling Sheath; 5: Mature Root; 6: Mature Stem; 7: Mature Leaves; 8: Mature Flower; 9: Mature Endosperm; 10: dried seeds; 11: wet seeds

To investigate *Os6* expression in rice, we isolated various tissues and organs from WT rice plants at different developmental stages. RNA was extracted and *Os6* expression was confirmed in various tissues and organs using qRT-PCR. *Os6* was widely expressed to different degrees in various tissues and organs (Fig. 3), with a relatively high expression level in seedlings and a relatively low expression level in mature leaves. Then, the rice glycosyltransferase gene *Os6* was successfully cloned from rice cDNA (Additional file 1: Figure S1a).

### Construction of the prokaryotic expression vector and the Os6 protein’s activity

**Fig. 4:**
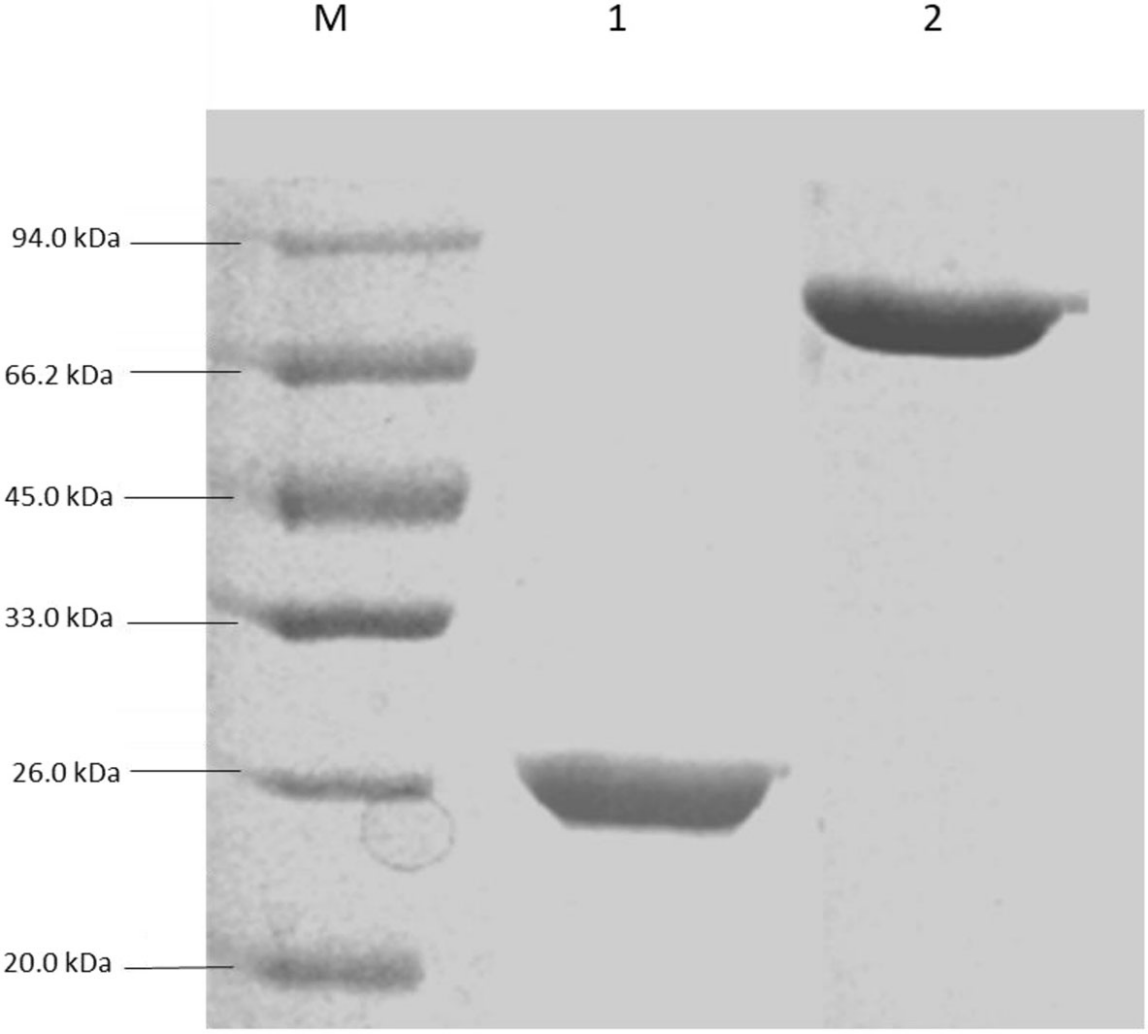
The activity of the OS6 protein was obtained. In order to obtain the Os6 protein, the protein was extracted from protein expression strain and seperated by protein SDS-PAGE gel

After sequencing, the *Os6* gene was cloned into a prokaryotic expression vector, PGEX (Additional file 1: Figure S1b). To investigate the Os6 protein’s activity, we cloned the target gene into the PGEX vector, and then transformed it into the *E. coli* protein-expression strain XL1-Blue. Protein expression was induced and confirmed by sodium dodecyl sulfate-polyacrylamide gel electrophoresis (Fig. 4).

### Rice glycosyltransferase Os6’s enzymatic reaction and substrate identification

**Fig. 5:**
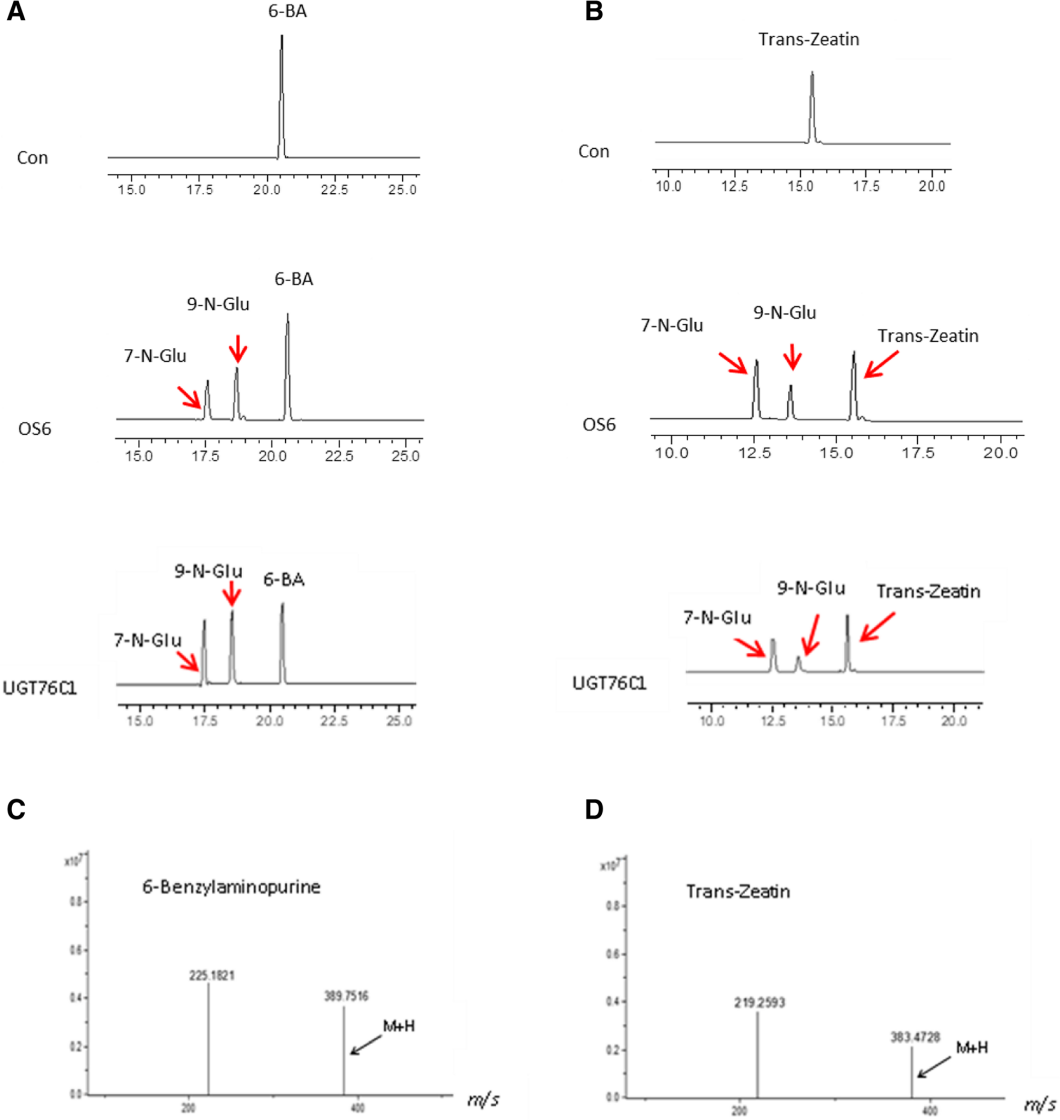
Rice glycosyltransferase OS6 enzymatic reaction and substrate identification. OS6 can transfer glucose to two cytokinins, trans-zeatin and 6-Benzylaminopurine, which were detected by HPLC and LC/MS mass spectrometry. **a**. Rice glycosyltransferase Os6 can glycosyl 6-BA by HPLC. **b**. Rice glycosyltransferase Os6 can glycosyl tZ by HPLC. **c**. Rice glycosyltransferase Os6 can glycosyl 6-BA by LC/MS. **d**. Rice glycosyltransferase Os6 can glycosyl tZ by LC/MS

To investigate whether Os6 could glycosylate the plant hormone cytokinin, we tested this enzymatic reaction in vitro by mixing Os6 protein, cytokinin, buffer and UDP-glucose. The products of the reaction mixture were examined using HPLC, and Os6 can transfer glucose to two cytokinins, trans-zeatin and 6-benzylaminopurine (Fig. 5a, b). The Arabidopsis glycosyltransferase gene UGT76C1 was used as a positive control. LC-MS was used to confirm the presence of glycosylated cytokinins produced by Os6 activity (Fig. 5c, d).

### Evaluation of the K_m_ values of Os6

Having found that Os6 could glycosylate cytokinins, we qualitatively analyzed the K_m_ values of these cytokinin reactions using HPLC (Table 1). The K_m_ values for Os6-mediated glycosylation of trans-zeatin and 6-benzylaminopurine were similar to those of UGT76C1.

**Table 1:** The Km values of OS6, which glycosylate trans-zeatin and 6-Benzylaminopurine

Substrates		Specific activity (nkat/mg protein)			
		76C1		Os6	
		7-N	9-N	7-N	9-N
Trans-Zeatin		1.58	0.61	1.79	0.85
6-Benzylaminopurine		0.95	1.53	0.68	1.22

### *Os6* overexpression in Arabidopsis

**Fig. 6:**
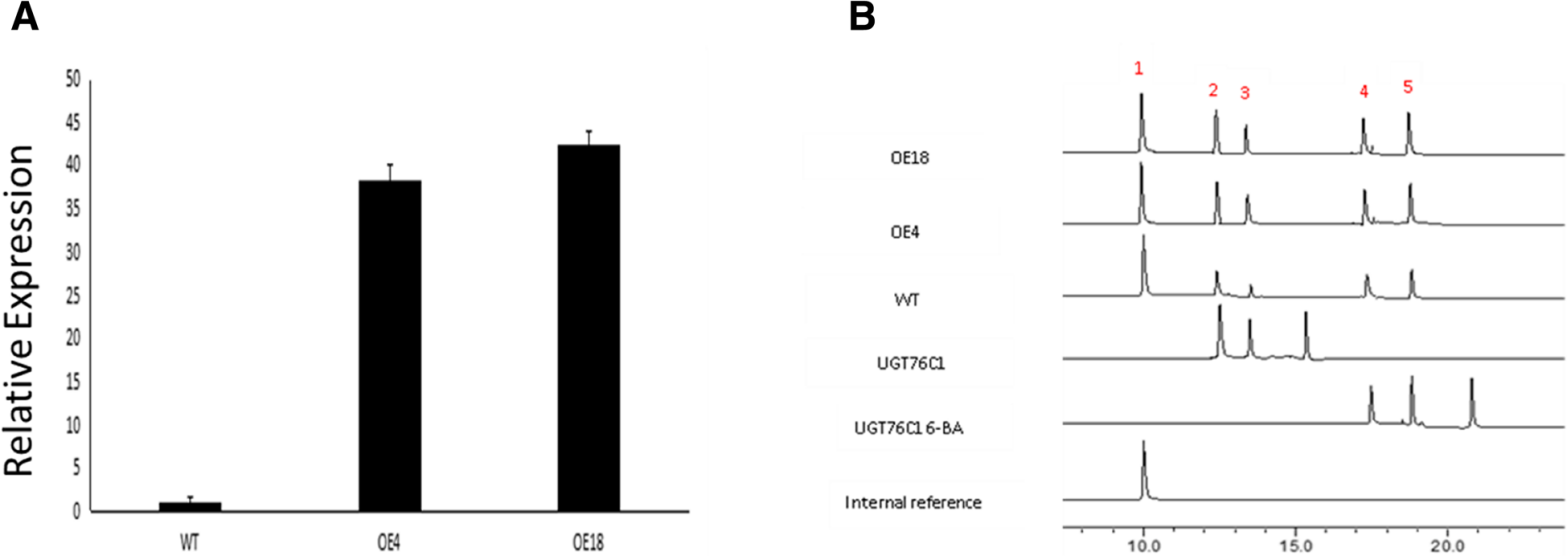
Overexpression *Os6* into Arabidopsis and determination their cytokinin glycosides. Overexpression of rice glycosyltransferase gene *Os6* into Arabidopsis and determination of cytokinin glycosides in transgenic Arabidopsis. **a**. The relative expression of *Os6* in Arabidopsis. **b**. The content of cytokinin glycosides in transgenic Arabidopsis

To determine whether Os6 is functionally active in plants, we overexpressed *Os6* in the model plant Arabidopsis. After three generations of screening, we obtained 25 transgenic homozygous lines. RNA was extracted and reverse transcribed into cDNA, and the expression of *Os6* in each line was confirmed by qRT-PCR. Two highly overexpressing strains, OE4 and OE18, were selected for subsequent experiments (Fig. 6a).

### Cytokinin glycoside measurements in transgenic Arabidopsis

The WT and transgenic Arabidopsis plants were grown under normal conditions for 2 weeks, after which the cytokinins and their glycosides were extracted using an organic solvent. Esculetin was used as an internal standard to quantitatively analyze each line by HPLC. The cytokinin glycoside content in the OE4 and OE18 overexpressing lines was greater than in the WT plants (Fig. 6b). Thus, the rice glycosyltransferase Os6 appears to glycosylate cytokinins *in planta*, and Os6 may act in the cytokinin pathway.

## Discussion

In our research, first, we compared database information based on known conditions, and constructed a phylogenetic tree based on sequence similarity using MEGA software. The results are shown in Fig. 1. Owing to the lengths of the sequenced genes, the differences are large. The data analysis results showed obvious branches, but most rice glycosyltransferase genes were not associated with the already published cytokinin glycosyltransferase gene UGT76C1 and UGT76C2 in Arabidopsis (Smehilova et al. 2016) (Additional file 1: Figure S2). In our study, the eight studied rice cytokinin glycosyltransferase genes were randomly selected.

Because the other seven candidate genes are not glycosylated cytokinins, only Os6 can glycosylate cytokinins. Then, we compared the gene sequence of *Os6* with the genes of the Arabidopsis glycosyltransferase gene family members. However, there is no sequence homologous to *Os6* in Arabidopsis. We also compared the sequence of *Os6* with the sequences of UGT76C1 and UGT76C2, and found that the similarity was ~ 40%. After the analysis, we found that, in addition to the conserved 44 amino acids at the C-terminus of glycosyltransferase, there are other highly similar gene sequences, and we speculate that these sequences enable these genes to glycosylate cytokinins.

Here, we cloned a glycosyltransferase gene from rice and constructed a prokaryotic expression vector to purify the active enzyme protein. After adequate incubation, an in vitro enzymatic reaction was detected by HPLC. The rice glycosyltransferase Os6 was able to glycosylate cytokinins. Glycosyltransferase proteins that can glycosylate cytokinins have been described previously. In 2004, Hou et al. found that Arabidopsis UGT76C1 and UGT76C2 can glycosylate cytokinins (Hou et al. 2014); however, since then no new proteins have been reported. In this research, we identified a new protein, Os6, that can glycosylate cytokinins. The K_m_ value of Os6 is similar to that of UGT76C1. Therefore, we propose that Os6 in rice has the same function as UGT76C1 in Arabidopsis. In the future we will try to generate transgenic rice overexpressing *Os6* to study its function in rice.

The glycosyltransferase Os6 is the first protein to be identified from rice that can glycosylate cytokinins. With the completion of the rice genome, the functions of many rice genes have been determined. However, most of the genes in the rice GT1 family have not yet been identified. There have been limited studies on cytokinin synthesis in rice and, in particular, the downstream pathway of cytokinin glycosylation. Moreover, there have been no new discoveries regarding cytokinin glycosyltransferases in rice. In this project, we identified eight candidate cytokinin glycosyltransferases genes and used LC-MS to determine whether they were functionally active in glycosylating cytokinins. Our results will allow future researchers to investigate the role of the Os6 cytokinin glycosyltransferase in rice.

*Os6* was overexpressed in Arabidopsis, and transgenic lines were obtained through three generations of screening. Measurements of the cytokinin glycosides produced by these transgenic lines showed that Os6 is functionally active as a glycosylase in plants. Thus, Os6 is part of the cytokinin metabolic pathway in plants. However, because this study was carried out in the model plant Arabidopsis and involves heterologous expression, our results may not be transferable to rice. Therefore, in future, we plan to examine the function of Os6 in transgenic rice.

A spatiotemporal expression analysis of rice plants showed that *Os6* is highly expressed in young organs and in leaf sheaths. This suggests that Os6 may play a specific role in these tissues. Transgenic rice overexpressing *Os6* will be useful for examining the growth and development of these parts and the distributions of cytokinins.

Cytokinins are one of the five main plant hormones and play very important roles in plants (Larrieu et al. 2015). Here, we examined cytokinin glycosylation and the cytokinin contents in plants. However, cytokinins have important relationships with other plant hormones, including auxin and abscisic acid, that also affect growth and development (Li et al.^2016^). Therefore, research on these hormones may also prove to be important in the future.

## Conclusion

In this study, we cloned the rice glycosyltransferase gene *Os6*, constructed a prokaryotic expression vector, and expressed and purified the active enzyme protein in bacteria. Using in vitro enzymatic reactions and LC-MS, we found that Os6 can glycosylate cytokinins in vitro. We then overexpressed *Os6* in Arabidopsis and extracted cytokinin glycosides from transgenic and WT plants. The transgenic Arabidopsis plants had increased cytokinin glycosides in comparison with the WT-plants, indicating that Os6 can also glycosylate cytokinins *in planta* and may be involved in the cytokinin pathway of rice.

## Methods

### Plant materials and growth conditions

The rice Nipponbare ecotype was used in this study. Intact and uniformly sized rice grains were surface sterilized with 75% ethanol for 2 min, 0.1% mercuric chloride solution for 3 min, and then washed 3–4 times with sterile water. The seeds were placed in sterile culture bottles containing a small amount of water for 3 d until germination. The seedlings were then transferred to plastic pots and grown in a greenhouse at 30 °C with natural light (12 h light and 12 h dark).

Arabidopsis ecotype Columbia was used as a plant model in this study. Mature intact wild-type (WT) seeds were sterilized with 0.1% Hg for 2 min, 75% alcohol for 3 min, and then rinsed 3–5 times with sterile water. The seeds were placed in Murashige and Skoog medium, vernalized at 4 °C in the dark for 3 d, and then germinated in a culture chamber at 22 °C with 16 h light (100 μmol m^− 2^ s^− 1^) and 8 h dark for 2 weeks. The seedlings were then transplanted to small pots containing soil and grown under culture chamber conditions as described.

### RNA extraction and reverse transcription

For the RNA extraction, young WT rice plants at the two-leaf and one-heart stage were ground to a powder in liquid nitrogen and 1 mL of TRIzol reagent was added per 0.2 mg of powder. The sample was then mixed by inversion and, after standing for 5 min, 200 μL phenol chloroform was added. The sample was vortexed to denature the plant proteins and, after standing for 5 min, was centrifuged at 13,400×*g* for 5 min at 4 °C. The supernatant was transferred to a new centrifuge tube, an equal volume of isopropanol was added, and the sample was mixed by inversion. After standing for 10 min, the sample was again centrifuged for 5 min at 4 °C, the supernatant discarded, and 1 mL of 75% ethanol was added. The flocculated precipitate was washed, and then centrifuged at 13,400×*g* for 1 min at 4 °C. The ethanol was discarded, the sample was centrifuged at 13,400×*g* for 1 min at 4 °C and the liquid carefully removed using a 20-μL pipette. The sample was air dried at room temperature for 5 min and 20–50 μL DEPC water was added to dissolve the nucleic acids. Samples were stored at − 20 °C until required. For the reverse transcription, a reverse transcription kit (TaKaRa, Japan) was used according to the manufacturer’s protocol.

### Quantitative RT-PCR

For the first step of the quantitative real-time PCR (qRT-PCR), total RNA was extracted from 2-week-old rice plants using TRIzol reagent. For the second step, reverse transcription from RNA to cDNA, a Primescript RT reagent kit (TransGen, TaKaRa) was used. A 20 μL reaction volume was used for the qRT-PCR, which was performed using a Bio-Rad real-time thermal cycling system. SYBR-Green (TaKaRa) was used to detect quantitative gene expression, with ubiquitin used as an internal control. The data were analyzed using Bio-Rad CFX Manager software. The primer information is included in Additional file 2: Table S1.

### Plasmid construction and plant transformation

1. Candidate gene cloning. RNA was extracted from WT rice and reverse-transcribed into cDNA. Candidate gene sequences were amplified using the primers F:CGCGGATCCATGACAGCACCGATGAC and R:CGGGGTACCCTAGTGTTCTTCCACTC, which were designed using the sequences available on the rice genome website. After amplification, the candidate gene fragment was ligated into an intermediate carrier and its sequence was confirmed by sequencing.
2. Construction of prokaryotic expression vectors containing candidate genes. To facilitate cloning we modified a common prokaryotic expression vector, PGEX-2 T (Zhang et al. 2008), by inserting a multiple-cloning site between the BamHI and EcoRI restriction sites (BamHI-NdeI-NotI-SphI-NcoI-SalI-SacI-XhoI-HindIII-EcoRI) and renamed the vector pGEX-3H. Each rice gene was individually cloned into pGEX-3H and the plasmid isolated. After verification by sequencing, the plasmid was transformed into the *Escherichia coli* expression strain XL1-Blue, to express the fusion protein OS6-glutathione S-transferase.

### Purification of cytokinin glycosyltransferase protein and in vitro cytokinin glycosyltransferase enzymatic reactions

(i) Protein expression was induced in XL1-Blue by incubating the bacteria overnight at 37 °C and then culturing them to an OD_600_ = 0.8–1.0 at 20 °C with shaking at 180 rpm. Isopropyl β-D-thiogalactoside was added to induce the expression of the protein encoded by the rice gene. (ii) To isolate the target protein, the bacterial cells were collected by centrifugation at 5000×*g* for 10 min at 4 °C. The cells were resuspended in phosphate buffer saline and disrupted by sonification. The solution was applied to a column to isolate the target protein using its glutathione S-transferase tag. A protease was used to elute the protein and obtain a high purity level for the enzyme activity analysis. (iii) For the in vitro enzymatic reactions, 2 μg UGT-purified protein was added to a 100-μL reaction mixture containing 1 mM cytokinin, 5 mM UDP-glucose, 50 mM *N*′-α-hydroxythylpiperazine-*N*′- ethanesulfanic acid buffer (pH 7.0) and 14.4 mM 2-mercaptopethanol and incubated at various temperatures for different reaction times.

### High-performance liquid chromatography (HPLC) detection of substrates and (K_m_) value determinations

After the enzymatic reaction, the mixture was filter-sterilized and added to a 5-μm C18 HPLC column (150 × 4.6 mm Zorbax; Agilent, USA) and gradient-eluted at room temperature. Phase A was a filter-sterilized organic solvent in methanol and Phase B was a filter-sterilized 0.1% phosphoric acid aqueous solution. These were applied to the column as follows: 90% Phase A and 10% Phase B for 22 min, 35% Phase A and 65% Phase B for 25 min, and 90% Phase A and 10% Phase B for 35 min. An HPLC spectrophotometer (Shimadzu, Japan) was used with a D2 and set to a 270-nm detection wavelength. For the MS, we used a Shimadzu LC-MS system. The column used was the same as that used for the LC. The Phase A solution was still methanol, while the Phase B solution was 0.1% formic acid. The heater was set to 180 °C and the mass spectrometer was operated with a positive electrospray ionization mode of 50 eV and a 5.0-kV probe voltage. Data acquisition and analysis was performed using Xcalibur software (version 2.0.6).

To calculate the K_m_ of the glycosyltransferase and its substrate, a Michaelis-Menten kinetic analysis was performed. We used the same reaction system as described above. The reaction was performed in a 30 °C water bath for 1, 5, 10, 15, 20, 30, 40, 60, 90, 120 and 180 min. Reactions were snap-frozen in liquid nitrogen and either used immediately for HPLC or were stored at 20 °C until required.

To determine the kinetic constants, nine concentration gradients were prepared using substrate concentrations of 0–1 mM, 0.025 mM, 0.05 mM, 0.075 M, 0.1 mM, 0.15 mM, 0.2 mM, 0.4 mM, 0.6 mM, 1.0 mM or 2 μg/mL. The proteins being tested were incubated with the substrate and 5 mM UDP-glucose in the optimum buffer at the optimum pH, temperature and time. The final HPLC results were analyzed and the most suitable K_m_ and maximum rate (V_max_) values were calculated from the kinetic equations.

### Generation of transgenic Arabidopsis and cytokinin extraction

To generate the *Os6* overexpression lines, we cloned the full-length *Os6* cDNA from rice into the pBI121 binary vector, which is driven by the CaMV35S promoter. This was then transformed into to the *Agrobacterium tumefaciens* GV3103 strain. Strain GV3103 containing the rice glycosyltransferase gene *Os6* was activated and grown to a concentration of approximately OD_600_ = 0.8. The cells were collected by high-speed centrifugation at 13,400×*g* for 10 min at 4 °C and diluted in 1% sucrose to OD_600_ = 0.1. Then, the Silwet L-77(GE, Beijing) activator was added, and the bacterial solution was used for in plant transformations at room temperature. WT Arabidopsis plants at 4–5 weeks of age were selected for transformation because of their vigorous growth and stage (flowering) in which the stem is 1 cm in length but the anther has not yet grown. The flower buds were dipped into the Agrobacterium GV3103 solution for 15 s three times. The plants were then covered with black cloth for 24 h to allow infection to occur. After flower maturation and seed formation, the seeds were collected, and transformants were selected on medium containing kanamycin. Homozygous transgenic Arabidopsis plants overexpressing *Os6* were obtained after three generations of antibiotic selection.

To extract cytokinins from the plant tissues, 2-week-old WT and transgenic Arabidopsis plants were independently ground in liquid nitrogen. Then, 80% methanol solution was added, and the samples were mixed by inversion at 4 °C for 24 h. The mixtures were centrifuged at 13,400×*g* for 10 min and the supernatant were collected. The mitogen glycoside solutions were isolated using the described cytokinin HPLC protocol, with UGT76C1 as a positive control.

## Additional files

Additional file 1: **Figure S1.** Cloning and Construction of the Prokaryotic Expression Vector. The cloning of Rice Glycosyltransferase gene OS6 and the constructing of its prokaryotic expression vetor. A. Cloning Rice Glycosyltransferase gene Os6 from rice gene data bank. B. Constructing of prokaryotic expression vetor of rice glycosyltransferase gene Os6. **Figure S2.** Phylogenetic tree comparison of UGT76C1 and UGT76C2 with rice cytokinin glycosyltransferase. To further understand the distant relationship of the rice glycosyltransferase phylogenetic tree, we compared the glycosyltransferase UGT76C1 and UGT76C2, which has been shown to be a glycosyl cytokinin in Arabidopsis, to the predicted cytokinin glycosyltransferase phylogenetic tree in rice. (DOCX 9898 kb)

Additional file 2: **Table S1.** Primers used in this study. (DOC 31 kb)

## Abbreviations

APRRs: Arabidopsis pseudoresponse regulators
ARRs: Arabidopsis response regulators
CKX: Cytokinin oxidative dehydrogenase
CRE1: Cytokinin response 1
DMAPP: Dimethyl propylene diphosphate
GST: Goods and Services Tax
GT1: Family 1 glycosyltransferases
HEPES: N′-ɑ-hydroxythylpiperazine-N′-ethanesulfanic acid
iPA: Isoamyladenosine
iPDP: Isoamyladenosine-5-diphosphate
iPMP: Adenosine-5-phosphate
IPTG: Isopropyl β-D-Thiogalactoside
iPTP: Isoprenoid adenosine-5-triphosphate
LC-MS: Liquid chromatography-mass spectrometry
MS: Murashige and skoog
PBS: Phosphate buffer saline
qRT-PCR: Quantitative real-time PCR
RT: Room temperature
SDS-PAGE: Sodium dodecyl sulfate polyacrylamide gel electrophoresis
tRNA: Transport RNA
WT: Wild-type
ZMP: Trans-zeatin nucleoside phosphate
