# Supplementary material for: Identification and characterization of the first cytokinin glycosyltransferase from rice
Source: Rice (N Y). 2019 Mar 29;12:19. doi: 10.1186/s12284-019-0279-9 (PMC6439077; doi:10.1186/s12284-019-0279-9)
Supplement: Supplementary file 1 — Figure S1. Cloning and Construction of the Prokaryotic Expression Vector. The cloning of Rice Glycosyltransferase gene OS6 and the constructing of its prokaryotic expression vetor. A. Cloning Rice Glycosyltransferase gene Os6 from rice gene data bank. B. Constructing of prokaryotic expression vetor of rice glycosyltransferase gene Os6. Figure S2. Phylogenetic tree comparison of UGT76C1 and UGT76C2 with rice cytokinin glycosyltransferase. To further understand the distant relationship of the rice glycosyltransferase phylogenetic tree, we compared the glycosyltransferase UGT76C1 and UGT76C2, which has been shown to be a glycosyl cytokinin in Arabidopsis, to the predicted cytokinin glycosyltransferase phylogenetic tree in rice. (DOCX 9898 kb) [file 12284_2019_279_MOESM1_ESM.docx]

**Figure S1**

(A) (B)

1500bp

4000bp

1500bp

M OS6-PGEX OS6-PGEX


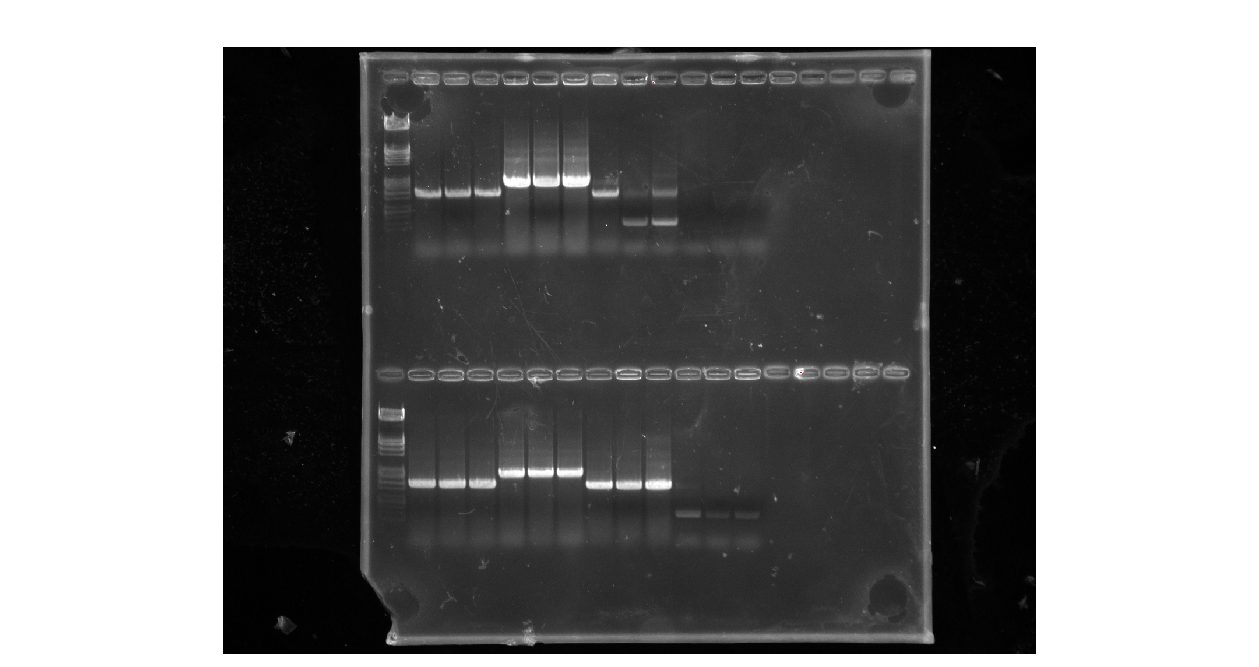

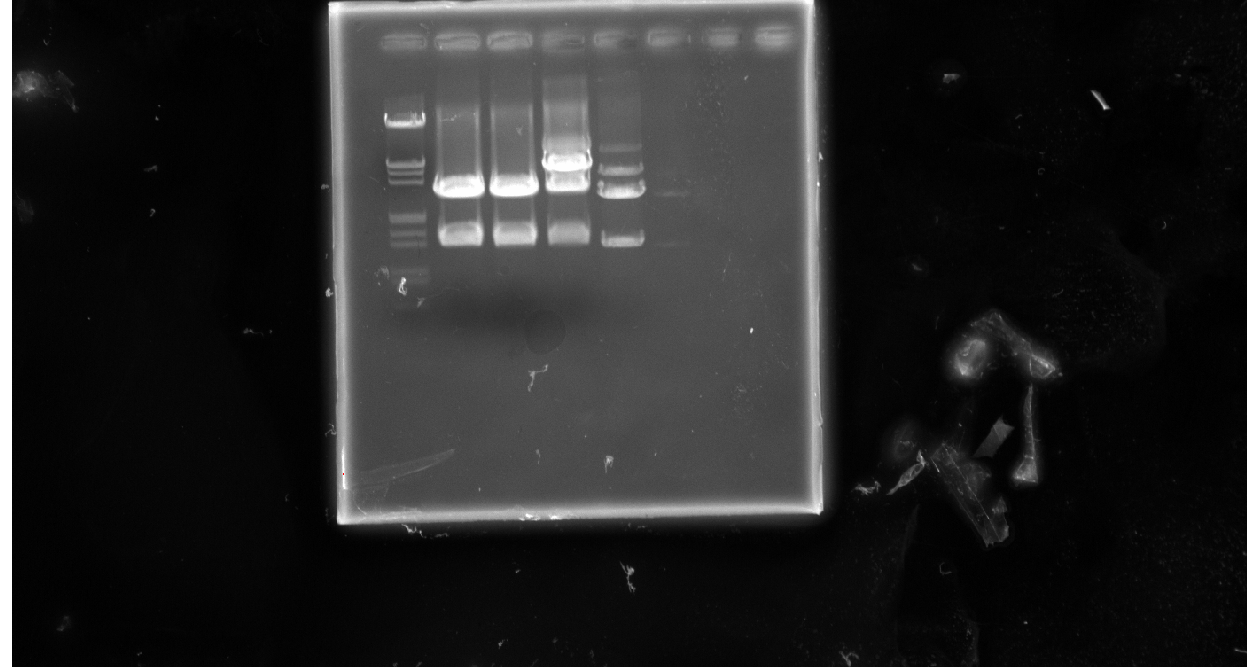


M OS6 OS6-PGEX

**Figure S2**

Figure S1

Cloning and Construction of the Prokaryotic Expression Vector

The cloning of Rice Glycosyltransferase gene OS6 and the constructing of its

prokaryotic expression vetor

A. Cloning Rice Glycosyltransferase gene Os6 from rice gene data bank.

B. Constructing of prokaryotic expression vetor of rice glycosyltransferase gene Os6

Figure S2

Phylogenetic tree comparison of UGT76C1 and UGT76C2 with rice cytokinin glycosyltransferase

To further understand the distant relationship of the rice glycosyltransferase phylogenetic tree, we compared the glycosyltransferase UGT76C1 and UGT76C2, which has been shown to be a glycosyl cytokinin in Arabidopsis, to the predicted cytokinin glycosyltransferase phylogenetic tree in rice.
