# Supplementary material for: Identification and characterization of the first cytokinin glycosyltransferase from rice
Source: Rice (N Y). 2019 Mar 29;12:19. doi: 10.1186/s12284-019-0279-9 (PMC6439077; doi:10.1186/s12284-019-0279-9)
Supplement: Supplementary file 2 — Table S1. Primers used in this study. (DOC 31 kb) [file 12284_2019_279_MOESM2_ESM.doc]

**Table S1. Primers used in this study.**

| **Primer names** | **sequence (5'-3')** |
| --- | --- |
| **For amplifying full-length cDNA** | |
| **Os6-F** | **CGCGGATCCATGACAGCACCGATGAC** |
| **Os6-R** | **CGGGGTACCCTAGTGTTCTTCCACTC** |
| **UGT76C1-F** | **CGCGGATCCGCCGCCATGGAGAAGAGAAACGAGAGAC** |
| **UGT76C1-R** | **CGGGAGCTCTCACGTAGGCACTAGTGGCT** |
| **For real-time PCR** |  |
| **RT-Os6-F** | **TCCATGACAGCACCGATGAC** |
| **RT-Os6-R** | **TACCCTAGTGTTCTTCCACTC** |
